# Effectiveness and safety of blonanserin in young and middle-aged female patients with schizophrenia: data from a post-marketing surveillance

**DOI:** 10.1186/s12888-023-04598-y

**Published:** 2023-02-21

**Authors:** Qijing Bo, Xijin Wang, Xuejun Liu, Hong Sang, Zhiyuan Xun, Ruiling Zhang, Xiaodong Yang, Huaili Deng, Keqing Li, Jindong Chen, Meijuan Sun, Guijun Zhao, Xianglai Liu, Duanfang Cai, Guilai Zhan, Juhong Li, Haiyun Li, Gang Wang

**Affiliations:** 1 The National Clinical Research Center for Mental Disorders & Beijing Key Laboratory of Mental Disorders & Beijing Institute for Brain Disorders Center of Schizophrenia, Beijing Anding Hospital, Capital Medical University, Beijing, 100088 China; 2 Department of Psychiatry, The First Psychiatric Hospital of Harbin, Harbin, Heilongjiang 150010 China; 3 Department of Psychiatry, Brain Hospital of Hunan Province, Changsha, Hunan 410007 China; 4 Mental Health Center, Changchun Sixth Hospital, Changchun, Jilin 130052 China; 5 Department of Psychiatry, Tianjin Anding Hospital, Tianjin, Tianjin, 300222 China; 6 Department of Psychiatry, Henan Mental Hospital, Xinxiang, Henan 453002 China; 7 Department of Psychiatry, Shandong Mental Health Center, Jinan, Shandong 250014 China; 8 Department of Psychology, Psychiatric Hospital of Taiyuan City, Taiyuan, Shanxi, 030000 China; 9 Department of Psychiatry, Hebei Provincial Mental Health Center, Baoding, Hebei 071000 China; 10 Department of Psychiatry, National Clinical Research Center for Mental Disorders, The Second Xiangya Hospital of Central South University, Changsha, Hunan 410011 China; 11 Department of Pharmacy, Daqing Third Hospital, Daqing, Heilongjiang 163712 China; 12 Department of Psychiatry, Guangyuan Mental Health Center, Guangyuan, Sichuan 628001 China; 13 Institute of Mental Health, Hainan Provincial Anning Hospital, Haikou, Hainan, 570206 China; 14 Department of Psychiatry, The Fifth People’s Hospital of Zigong, Zigong, Sichuan 643020 China; 15 Department of Psychiatry, Xuhui Mental Health center, Shanghai, 200232 China; 16 Department of Psychiatry, The Fourth People’s Hospital of Chengdu, Chengdu, Sichuan 610036 China; 17 Medical Affairs, Sumitomo Pharma (Suzhou) Co., Ltd, Shanghai, 200025 China; 18 Advanced Innovation Center for Human Brain Protection, Capital Medical University, Beijing, 100088 China; 19 The National Clinical Research Center for Mental Disorders & Beijing Key Laboratory of Mental Disorders, Beijing Anding Hospital, Capital Medical University, Beijing, 100088 China

**Keywords:** Blonanserin, Effectiveness, Safety, Women, Schizophrenia

## Abstract

**Background:**

A post-marketing surveillance of blonanserin has been ongoing since September 2018. The aim of this study was to assess the effectiveness and safety of oral blonanserin in Chinese young and middle-aged female patients with schizophrenia in real clinical settings, using the data from the post-marketing surveillance.

**Methods:**

A 12-week, prospective, multi-center, open-label, post-marketing surveillance was conducted. Female patients aged 18–40 years were included in this analysis. The Brief Psychiatric Rating Scale (BPRS) was used to evaluate the effectiveness of blonanserin in improving psychiatric symptoms. The incidence of adverse drug reactions (ADRs) such as of extrapyramidal symptoms (EPS), prolactin elevation and the weight gain were used to evaluate the safety profile of blonanserin.

**Results:**

A total of 392 patients were included both in the safety and full analysis sets, 311 patients completed the surveillance protocol. The BPRS total score was 48.8 ± 14.11 at the baseline, decreasing to 25.5 ± 7.56 at 12 weeks (*P* < 0.001, compared with baseline). EPS (20.2%) including akathisia, tremor, dystonia, and parkinsonism were found as the most frequent ADRs. The mean weight gain was 0.27 ± 2.5 kg at 12 weeks from the baseline. Four cases (1%) of prolactin elevation were observed during the period of surveillance.

**Conclusion:**

Blonanserin significantly improved the symptoms of schizophrenia in female patients aged 18–40 years; the drug was well tolerated and had a low tendency to cause metabolic side effects, including prolactin elevation in these patients. Blonanserin might be a reasonable drug for the treatment of schizophrenia in young and middle-aged female patients.

## Introduction

Schizophrenia is a serious mental disorder that affects the person’s thinking, feeling, and behavior. Gender differences have long been discussed, including the variations in the symptom profile, neurobiology, treatment responses, and side effects from the treatment in female and male patients with schizophrenia [1]. Female patients with schizophrenia are older at the onset of illness, have better premorbid adjustments and intelligence quotients, which benefit the initial treatment [2]. The pregnancy and the postpartum periods are unique in the course of life for women, during which, the treatment needs to be adjusted [3]. Although female patients respond well to lower doses of antipsychotic medications, rates of side effects to many drugs are higher in women than men, [4] which could be explained by higher serum levels of these drugs in women [5]. Especially, prolactin levels and BMI/weight gain are higher in female patients than in male patients with schizophrenia spectrum disorder, [6] treated with a lower dosage of antipsychotic drugs [7]. Additionally, female patients with schizophrenia are more likely to have physical comorbidities than men [8].

Difference in treatment responses between male and female, which might be due to biological, physiological and sociocultural factors, should be taken into account when antipsychotics are prescribed [9]. A previous study confirmed that women with schizophrenia suffered from some critical problems of physical health and reproductive health, and therefore, optimal treatment in women with schizophrenia was required [10]. The intervention in women patients with schizophrenia must focus on physical, mental, and social wellbeing beyond treating the symptoms [11]. Some scholars suggested that clinical practice guidelines for women with schizophrenia should integrate with gender-related recommendations, and further research needs to focus on the efficacy and tolerability of antipsychotic drugs, particularly in women [8, 12]. Women patients with schizophrenia are prescribed gender-specific drugs and doses differently by clinicians [13].

Blonanserin is an atypical antipsychotic agent, synthesized in the early 1980s. Blonanserin was shown to have potent antagonist properties against dopamine D_2_, D_3_ and serotonin 5-HT_2_ receptors, while it exhibited a low affinity to adrenaline α_1_, histamine H_1_, and muscarinic M_1_ receptors, using an in vitro receptor binding test [14, 15]. Furthermore, the brain/plasma concentration ratio of blonanserin in patients with schizophrenia was 3.38 times, indicating its good blood-brain barrier permeability [16]. All these pharmacological characteristics were associated with a relatively lower risk of possible complications during the treatment with the drug. Besides, blonanserin is mainly metabolized by cytochrome P450 (CYP) 3A4 in the liver [17]. Previous studies on healthy females showed that neither administration of contraceptive pills nor menopause or estrogen replacement therapy altered the intestinal or hepatic CYP3A activity [18, 19]. These qualities could suggest fewer potential drug interactions, and consequently, better drug adherence in women.

A phase 3, 8-week, double-blind, multicenter, randomized controlled study showed that its efficacy was comparable with haloperidol [20]. The previous and recent studies all showed effectiveness in schizophrenia with the blonanserin treatment compared with risperidone [21, 22]. A clinical trial showed that blonanserin as adjunct to previous treatment was comparable with olanzapine and repossessed relatively quick [23]. And a systematic review and meta-Analysis on efficacy, tolerability, and safety of blonanserin demonstrated that it had a good safety profile, with a low risk of side effects, such as weight gain and prolactin elevation [24]. In an epidemiological study, analysis of age-adjusted 12-month prevalence of mental disorders found that majority of schizophrenia patients were young and middle-aged; prevalence in females were higher than in males [25]. However, the relevant gender-specific research on this important young and middle-aged women population is limited. Therefore, the primary purpose of this study was to assess the effectiveness and safety of oral blonanserin in young and middle-aged female patients with schizophrenia in real-world clinical settings.

## Patients and methods

### Methods

A 12-week, prospective, multi-center, open-label, post-marketing surveillance was conducted. Patients with schizophrenia were recruited from September 2018 to May 2020 from 16 clinical sites across China. All patients taking blonanserin during the period were included and then followed up. Enrolled patients should orally receive 8–24 mg/day of blonanserin according to its approved dose and administration. The dose could be adjusted according to the treatment response and tolerability. The maximum daily dose is 24 mg. Female patients aged 18–40 years were included in this analysis. Efficacy and safety analyses were performed based on the full analysis set (FAS) and the safety set (SS). Both FAS and SS included all patients who received at least one blonanserin treatment. Electronic data capture (EDC) system collected the diagnosis and treatment information such as details on drug use, adverse reactions, and other related parameters including laboratory results and electrocardiograms.

The study protocols were approved by the ethics committees of the leading clinical site at the Second Xiangya Hospital of Central South University and the respective other study centers. Written informed consent was obtained or a waiver of informed consent was approved by the clinical site where the patients were enrolled.

### Drug effectiveness evaluation

The severity of schizophrenia was evaluated using the Brief Psychiatric Rating Scale (BPRS) [26, 27] at the baseline, 2/4 weeks, 6/8 weeks, and 12 weeks. The main outcome was the mean change in BPRS total score from the baseline to the end of treatment (day 1 as the baseline). The BPRS was an 18-item, 7-point rating system with a score for each item in the range 1–7, and a total score in the range of 18–126. The BPRS was divided into five factors: anxiety-depression (anxiety, guilt, depression, and somatic concern); anergia (emotional withdrawal, motor retardation, blunted affect, and disorientation); thought disturbance (conceptual disorganization, grandiosity, hallucinatory behavior, and unusual thought content); activation (tension, mannerisms and posturing, and excitement); hostility-suspiciousness (hostility, suspiciousness, and uncooperativeness). The 5-factor model scores were also determined.

### Safety evaluation

Adverse events (AEs) were coded according to the ICH International Dictionary of Medical Terms (MedDRA version 21.0, the Medical Dictionary for Regulatory Activities). AEs and adverse reactions (ADRs) during the treatment period were reported by the participating physicians. ADRs were defined as AEs whose causality to blonanserin could not be ruled out, as determined by the participating physicians.

### Statistical analysis

SAS version 9.4 software for Windows (SAS Institute, Cary, NC, USA) was used for the analysis. Categorical variables were shown as n (%) and continuous variables were summarized as mean ± standard deviation (SD). Paired t-test and analysis of variance (ANOVA) were used to compare continuous variables and χ^2^ analysis was used for categorical variables. The statistical significance level was defined with a two-tailed P-value of < 0.05.

## Results

### Baseline demographics and clinical characteristics

There were 408 female patients between the ages of 18 and 40 enrolled. Of these young and middle-aged female patients, 392 were included both in SS and FAS for this secondary analyses. And 311 (76.2%) patients completed the surveillance protocol. From the young and middle-aged female patients’ demographic data, the average age was 28.0 ± 6.2 years old (mean ± SD), and the average body weight was 61.5 ± 12.6 kg (mean ± SD). The average drug exposure duration was 75.3 ± 24.41 days (mean ± SD). The clinical characteristics are also shown in Table 1.

**Table 1:** Demographics of the young and middle-aged female patients with schizophrenia

Variates	Category	Blonanserin group (n = 392)
Age	Mean ± SD	28.0 ± 6.2
Height (cm)	Mean ± SD	163.1 ± 3.1
Weight (kg)	Mean ± SD	61.5 ± 12.6
Duration of illness (month)	Mean ± SD	29.8 ± 41.0
Baseline BPRS score	Mean ± SD	48.8 ± 14.1
Baseline diseases (n (%))	yes	30 (7.7)
Baseline medicine (n (%))	yes	205 (52.3)

BPRS = Brief Psychiatric Rating Scale

### Effectiveness of blonanserin

**Fig. 1:**
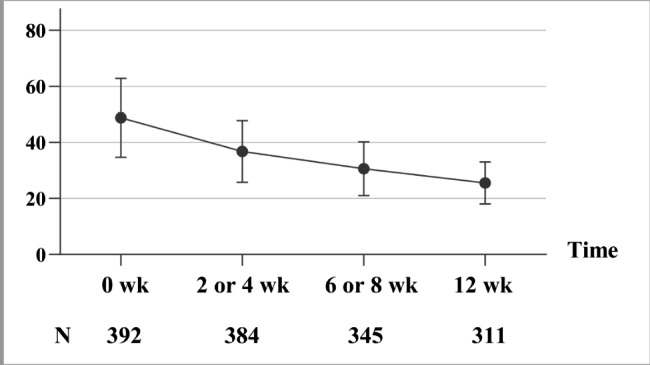
BPRS total score of young and middle-aged female patients with schizophrenia over time

In the effectiveness analysis, statistically significant differences were found between before treatment and after treatment within the group at each visit (P < 0.001). The BPRS total score was 48.8 ± 14.11 (N = 392; mean ± SD) at the baseline, decreasing to 36.8 ± 11.03 (N = 384; mean ± SD), 30.6 ± 9.63 (N = 345; mean ± SD), and 25.5 ± 7.56 (N = 311; mean ± SD) at 2/4, 6/8, and 12 weeks from the baseline, respectively (all P < 0.001 compared with the baseline). approximately 48.9% (152/311) of patients achieved a ≥ 50% decrease in BPRS total score from the baseline to week 12. The 5-factor model scores were also significantly lower in patients after 2/4 weeks of treatment than that of the baseline (P < 0.001) and continued to decrease thereafter (6/8 and 12 weeks from the baseline, Table 2). BPRS total score in young and middle-aged female patients with schizophrenia over time are shown in Fig. 1.

**Table 2:** BPRS total score and 5-factor model scores from the baseline to 12 weeks after initiation of treatment

BPRS (mean ± SD)	Baseline (N = 392)	2/4 weeks (N = 384)	6/8 weeks (N = 345)	12 weeks (N = 311)
Total Score	48.8 ± 14.1	36.8 ± 11.0^**^	30.6 ± 9.6**	25.5 ± 7.6^**^
Anxiety-depression	10.0 ± 3.5	8.1 ± 2.9^**^	7.0 ± 2.6^**^	5.9 ± 2.1^**^
Anergia	9.9 ± 3.5	7.9 ± 2.8^**^	6.7 ± 2.3^**^	5.9 ± 2.1^**^
Thought disturbance	11.9 ± 4.5	8.8 ± 3.6^**^	7.2 ± 3.2^**^	5.8 ± 2.3^**^
Activation	6.9 ± 3.2	5.0 ± 2.2^**^	4.2 ± 1.8^**^	3.7 ± 1.3^**^
Hostility-suspiciousness	10.1 ± 4.1	6.9 ± 3.0^**^	5.5 ± 2.5^**^	4.3 ± 1.8^**^

** P < 0.001, BPRS = Brief Psychiatric Rating Scale

### Safety of blonanserin

Among the 392 patients included, 92 (23.5%) patients developed ADRs. Subgroup analysis showed that 96 mild ADRs occurred in 58 patients (14.8%), among them, 71 mild ADRs occurred in 52 patients (13.3%) were classified as neurological disorders; forty-six moderate ADRs occurred in 33 patients (8.4%), of which 32 moderate ADRs occurred in 26 patients (6.6%) were classified as neurological disorders; one severe ADR occurred in 1 patient (0.3%) (Table 3).

**Table 3:** ADRs involving systematic and clinical manifestations (N = 392)

ADRs	Mild		Moderate		Severe	
	Number of patients, n (%)	Number of cases	Number of patients, n (%)	Number of cases	Number of patients, n (%)	Number of cases
Total ADRs	58 (14.8)	96	33 (8.4)	46	1 (0.3)	1
**Neurological disorders**	52 (13.3)	71	26 (6.6)	32	0 (0)	0
Akathisia	28 (7.1)	28	11 (2.8)	11	0 (0)	0
Tremor	17 (4.3)	18	5 (1.3)	5	0 (0)	0
Dystonia	7 (1.8)	7	5 (1.3)	6	0 (0)	0
Parkinsonism	8 (2.0)	9	4 (1.0)	4	0 (0)	0
**Investigations**	9 (2.3)	9	4 (1.0)	5	1 (0.3)	1
Weight gain	3 (0.8)	3	2 (0.5)	2	0(0)	0
Blood prolactin increased	2 (0.5)	2	1 (0.3)	1	1 (0.3)	1
Increased heart rate	1 (0.3)	1	1(0.3)	1	0(0)	0
elevated transaminase level	2 (0.5)	2	0 (0)	0	0 (0)	0
White blood cell count decreased	0 (0)	0	1 (0.3)	1	0 (0)	0
Blood glucose increased	1 (0.3)	1	0 (0)	0	0 (0)	0
**Gastrointestinal disorders**	2 (0.5)	3	2 (0.5)	2	0 (0)	0
**Eye disorders**	1 (0.3)	3	1 (0.3)	1	0 (0)	0
**Psychiatric disorders**	1 (0.3)	1	1 (0.3)	1	0 (0)	0
**Kidney and urinary disorders**	1 (0.3)	1	0 (0)	0	0 (0)	0

ADRs = Adverse reactions

Analysis of extrapyramidal symptoms.

**Fig. 2:**
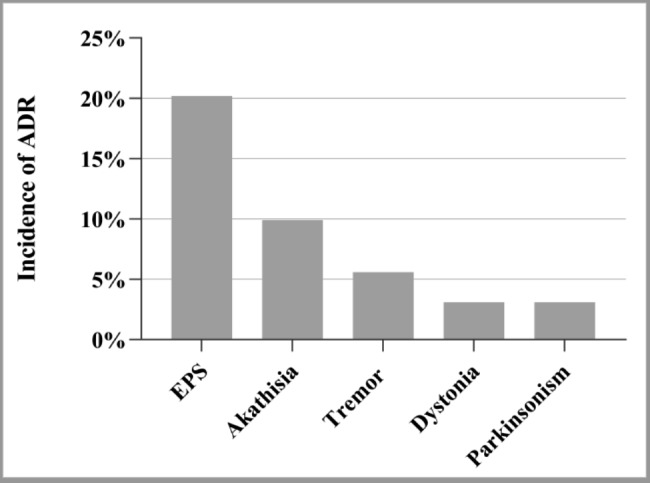
Frequent ADRs of young and middle-aged female patients with schizophrenia during the surveillance

Extrapyramidal symptoms (EPS) of ADRs occurred in 79 young and middle-aged females, with an incidence rate of 20.2%. Akathisia (9.9%), tremor (5.6%), dystonia (3.1%), and Parkinsonism (3.1%) were among the frequent ADRs. Frequently occurred ADRs during the surveillance are shown in Fig. 2.

Analysis of weight gain.

The average weight of the young and middle-aged female patients before treatment and week 12 were 61.53 ± 12.553 kg (mean ± SD) and 61.83 ± 12.069 kg (mean ± SD); the mean weight change was 0.27 ± 2.538 kg (mean ± SD), with no statistically significant difference in the intra-group comparison (P = 0.059). And 3.9% of patients gained weight ≥ 7% at week 12 from the baseline.

Analysis of prolactin elevation.

Four cases (1%) of prolactin elevation of ADR were observed during the surveillance.

### Blonanserin dosage

The average daily blonanserin dose during the treatment was 11.41 ± 4.31 mg (mean ± SD) among the 392 young and middle-aged female patients. A rising trend in the daily dose of blonanserin from week 1 (8.4 ± 3.05 mg/d, mean ± SD) to week 12 (12.2 ± 4.98 mg/d, mean ± SD) from the baseline was found.

## Discussion

In this analysis, we first assessed the effectiveness and safety of blonanserin in young and middle-aged female patients with schizophrenia, using post-marketing surveillance data. The results of this single-arm study showed that 12-week treatment of blonanserin effectively improved the clinical symptoms of young and middle-aged female patients with schizophrenia; Our study analysis also demonstrated good tolerability and safety of blonanserin by a low incidence of weight gain and hyperprolactinemia.

The young and middle-aged female patients with schizophrenia showed a significant improvement after blonanserin treatment. With the 12-week follow-up time, the BPRS score decreased gradually. In previous clinical trials, blonanserin, usually compared with risperidone and haloperidol, had comparable effectiveness [24, 28]. The research on Japanese post-marketing surveillances did not include subgroup analyses with different sexes and ages [29]. However, these post-hoc analyses confirmed the effectiveness of blonanserin in young and middle-aged female patients. A previous study elucidated the long-term blonanserin treatment for schizophrenia [30]. The current study had a follow-up of 12 weeks, but the results need to be verified in a longer course of illness.

Upon the exploration on the safety of 12-week blonanserin treatment in women patients with schizophrenia, most adverse events/reactions after taking the drug were mild to moderate, and the incidence of severe adverse events/reactions was relatively lower. The most common adverse reactions were EPS, including akathisia, tremor, dystonia, and parkinsonism. The incidence of EPS (20.2%) was higher than the incidence (2.4%) from Japanese post-marketing surveillance, while the EPS incidence of Japanese study did not include the incidence of akathisia (4.3%) or tremor (1.2%). [29]. Compared to the incidence of EPS (48.46%) from a phase 3 clinical trial in the Chinese population, which included the incidence of akathisia, the incidence of EPS in our study was lower [31]. However, the direct comparisons were not appropriate as the differences in dosage, the definition of EPS, and assessment requirement/instruments existed.

The incidence of akathisia was 9.9% in young and middle-aged female patients with schizophrenia in the current study. A review article summarizing 5 types of Japanese post-marketing surveillance showed that the incidence of akathisia among patients subjected to 12-week surveillance was 4.3% [29]. The differences in incidence of akathisia could attribute to the discrepancies in patients. Only young and middle-aged female patients were included in this subgroup analysis, while all patients with schizophrenia treating with blonanserin were analyzed in the review summarizing Japanese post-marketing surveillances. A recent review showed that incidence rate of akathisia with the second-generation antipsychotic drugs ranged from 2.9 to 13.0%, with a composite incidence of 3.7% [32]. Akathisia may cause patients distress, poor adherence, and an increased risk of suicide [33]. While the post-marketing surveillance in China did not distinguish between patients with or without adjunctive antipsychotic treatment, which may also be a possible reason for the higher incidence of akathisia. Clinicians should consider the risk of akathisia in clinical practice and use effective intervention for the treatment of acute antipsychotic-associated akathisia.

The results of the post-marketing surveillance of blonanserin showed that in the young and middle-aged female patients, only 3.9% of patients had a weight gain of more than 7% after taking blonanserin for 12 weeks, with no restrictions on adjunctive treatments potentially affecting the weight. The weight of patients with schizophrenia decreased at the last evaluation of the Japanese post-marketing surveillance of blonanserin, [29] which was similar to the phase 3 clinical trial conducted in China [31]. A systematic review and meta-analysis indicated that patients treated with olanzapine, quetiapine, and risperidone were more likely to suffer from weight gain as a side effect and experience clinically significant (more than 7%) weight gain [34]. A previous survey observed weight gain (more than 7%) in 24.1% of patients with olanzapine, 55.6% of patients with quetiapine, and 23.7% of patients with risperidone at week 12 from the baseline [35]. Furthermore, one year exposure of antipsychotics caused a clinically relevant weight-gain of more than 7% in approximately 80% of patients [36]. Blonanserin caused lower weight gain compared to other second-generation antipsychotic drugs.

Weight gain is a major health concern when taking antipsychotic drugs. Furthermore, the overweight is one of cardiovascular risk factors and a potential contributor to increased co-morbidity, such as diabetes and metabolism. The specific mechanism of development of weight gain by antipsychotic drugs has yet to be elucidated fully. It may be associated with the decreased activity caused by genetic and modulation of sedation and appetite caused by antagonism to serotonin or histamine receptors (5-HT_2c_ and H_1_) [37–39]. Blonanserin has a low or negligible affinity to 5-HT_2c_, H_1_, and M_1_ receptors, which may demonstrate the lower weight gain effect [21]. Antipsychotics-induced weight gain is more prevalent in female patients with schizophrenia [40]. Female patients are more weight conscious and being overweight may lead to social stigma and reduced adherence to the treatment. Blonanserin that has less impact on body weight and metabolic parameters could be chosen for female patients.

Individuals with antipsychotics-induced hyperprolactinemia may exhibit a wide range of clinical symptoms including galactorrhea, irregular menstruation, amenorrhea, gynecomastia and sexual dysfunction in the short term, and osteoporosis with the long-term treatment, which caused by antagonizing the D2 receptor [41]. Yet receptor binding has not explained all the differences between drugs. In general, drugs that cross the blood-brain barrier less effectively require higher serum concentrations to achieve the same striatal D_2_ receptor occupancy, thereby increasing the exposure of pituitary D_2_ receptors [42]. And a positron emission tomography study on patients with schizophrenia showed that blonanserin had good blood-brain barrier permeability, [16] suggesting a less exposure of pituitary to blonanserin. Among the second-generation antipsychotics, amisulpride, paliperidone, and risperidone provoked a significantly greater increase of prolactin [43]. In the previous clinical trials, blonanserin barely influenced the prolactin secretion, which was confirmed by the results of this study. Therefore, drugs like blonanserin with less impact on prolactin can be chosen for young and middle-aged women as better treatment.

The existence of gender differences in schizophrenia have been studied in many researches, while many aspects of that have not been conclusive. A previous study showed that the most subtype of schizophrenia is the paranoid subtype in women, [44] which indicated women patients have more positive psychotic symptoms. Meanwhile, women patients tend to have better cognitive function [44, 45]. Biological theories hold the protective role of estrogen in the dopaminergic system and specific neural circuits [45]. The effectiveness of blonanserin on psychotic symptoms, cognitive function, social function, daily living and subjective well-being were verified in previous and recent studies [46–48]. The current study has got the consistent conclusion, which is the effectiveness and safety of blonanserin in young and middle-aged female patients with schizophrenia.

Several study limitations should be noted. Since the current analysis using data from a post-marketing surveillance of blonanserin in the treatment of schizophrenia patients without a control group, a comparison with other antipsychotic drugs is not available. Besides, as being a real-world non-interventional study, quite a number of its patients did not provide their information about illness duration, history of pregnancy or childbirth etc., making it unlikely to further analyze the correlation between the age of onset, the course of disease and the effect of blonanserin, or the differences in the efficacy and safety of blonanserin in patients with or without a history of pregnancy or childbirth. Still, data collected in this study, although with some limitations, helped verify safety and effectiveness of blonanserin in young and middle-aged female patients with schizophrenia in normal clinical practice.

## Conclusion

Blonanserin significantly improved the clinical symptoms of schizophrenia in female patients aged 18–40 years; it was well tolerated and had a low propensity to cause metabolic side effects and hyperprolactinemia in these patients. Blonanserin is a better option for young and middle-aged female patients with schizophrenia. Further studies concerning gender-specific variations are needed to develop precise guidelines for the recommendation of treatment in schizophrenia.

## Data Availability

All data generated or analysed during this study are included in this published article.

## List of abbreviations

ADR: Adverse drug reaction
AE: Adverse events
ANOVA: Analysis of variance
BPRS: Brief Psychiatric Rating Scale
CYP: Cytochrome P450
EDC: Electronic data capture
EPS: Extrapyramidal symptoms
FAS: Full analysis set
SS: Safety set
SD: Standard deviation
